# Biosynthetic Machinery to Abiotic Stress-Driven Emission: Decoding Multilayer Regulation of Volatile Terpenoids in Plants

**DOI:** 10.3390/antiox14060673

**Published:** 2025-05-31

**Authors:** Yingying Shan, Songheng Jin

**Affiliations:** 1 Jiyang College, Zhejiang A&F University, Zhuji 311800, China; syy15968566686@163.com; 2 College of Landscape Architecture, Zhejiang A&F University, Hangzhou 311300, China

**Keywords:** volatile terpenoids, abiotic stress, biosynthesis, volatile emission

## Abstract

Volatile terpenoids (VTs) are key secondary metabolites that play dual roles as endogenous antioxidants and airborne signals in plants under abiotic stress. Their biosynthesis is orchestrated via the mevalonate (MVA) and 2-C-methyl-D-erythritol 4-phosphate (MEP) pathways, with metabolic plasticity regulated by transcription factors, phytohormonal crosstalk, and stress-responsive elements. Recent advances have revealed that VTs such as isoprene, monoterpenes, and sesquiterpenes help mitigate oxidative stress by scavenging reactive oxygen species (ROS) and modulating antioxidant enzyme systems. However, regulatory mechanisms of stress-induced VT emissions remain fragmented and species-dependent. This review synthesizes current knowledge of VT biosynthesis and emission under abiotic stress, highlights their antioxidant functions and regulatory architecture, and underscores their protective roles in redox homeostasis and stress signal transduction. By identifying key metabolic nodes (e.g., TPS, DXS and MYC2) and stress-responsive pathways, we propose potential molecular targets for the development of stress-resilient cultivars. The integration of VT-based traits into breeding strategies and production-oriented metabolic engineering offers promising avenues for improving crop performance, reducing oxidative damage, and supporting sustainable agricultural systems.

## 1. Introduction

Volatile terpenoids (VTs), a class of low-molecular-weight organic compounds biosynthesized as plant specialized metabolites, derive their molecular skeletons from the condensation of isoprene units (C_5_H_8_). This structurally diverse group encompasses monoterpenes (C_1__0_), sesquiterpenes (C_1__5_), and certain diterpenes, with approximately 55,000 identified members documented to date [1,2]. These compounds are widely distributed within plant secretory structures, including secretory cavities, resin ducts, laticifers, glandular trichomes, and epidermal tissues [3]. With their high volatility and chemical plasticity, VTs function as key mediators of plant–environmental interactions through airborne signaling networks.

The physiological functions of airborne VTs encompass dual-function defense mechanisms against biotic and abiotic stressors. In seed plants, VTs constitute species-specific floral and fruit scent profiles that mediate pollinator attraction and seed disperser recruitment, thereby ensuring reproductive success and evolutionary fitness [4,5]. Exemplifying this chemoevolutionary strategy, nocturnal moth-pollinated species such as *Clarkia breweri* emit linalool-enriched floral blends containing aromatic esters as key pollinator-targeted attractants [6]. Central to VTs’ functionality is their chemical defense arsenal, mediating direct toxicity and indirect resistance responses against herbivores and phytopathogens [7,8]. Notably, maize (*Zea mays*) plants under herbivore attack by *Diabrotica virgifera* larvae accumulate the sesquiterpene (E)-β-caryophyllene, which recruits entomopathogenic nematodes (*Heterorhabditis bacteriophora*) rather than directly deterring the pest, illustrating a sophisticated tripartite interaction among plants, herbivores, and nematodes [9]. Acting as airborne sentinels, VTs mediate interplant alarm signaling by triggering preemptive upregulation of jasmonate-dependent defense pathways in neighboring plants, thereby establishing community-wide resistance networks. A seminal study on spider mite-infested lima bean plants (*Phaseolus lunatus*) demonstrated that herbivore-induced VT emissions enable intact neighboring individuals to preemptively upregulate jasmonate-dependent defense pathways, significantly enhancing pest resistance [10]. Beyond their roles in biotic interactions, VTs enhance abiotic stress resilience via distinct biochemical mechanisms. Isoprene and monoterpenes stabilize thylakoid membrane fluidity under heat stress, preserving photosynthetic efficiency, while also functioning as phytoantioxidants to mitigate oxidative stress under drought conditions [11]. The commercial exploitation of VT diversity continues to expand, with monoterpenes (e.g., limonene and linalool) serving as core ingredients in the flavor and fragrance industries and the diterpenoid taxol remaining a frontline chemotherapeutic agent. Emerging evidence suggests that α-pinene emissions from coniferous forests exert potent anxiolytic effects through olfactory-mediated neuroendocrine modulation, inspiring advancements in forest-based phytotherapeutic applications.

VTs play multifaceted roles in both plant survival strategies and human societal development. Recent research has extensively studied VT emission rates and their spatiotemporal patterns, highlighting their dynamic regulation by biotic factors (e.g., plant ontogenetic stages and cross-kingdom interactions) and abiotic variables such as temperature gradients, relative humidity, seasonal fluctuations, and solar irradiance [12]. Once released into the atmosphere, VTs fulfill diverse physiological roles, ranging from ecological signaling to biochemical defense. VTs play a central role in plant responses to abiotic stress, functioning not only as endogenous antioxidants that regulate redox homeostasis but also as key mediators of signal transduction and environmental adaptation. At present, the enzymatic processes of the major biosynthetic pathways of VTs—namely the mevalonate MVA and MEP pathways—have been relatively well elucidated. However, their regulation by hormones and transcription factors remains highly species-dependent and exhibits considerable plasticity. Although the spatiotemporal dynamics of VT emission and their ecological signaling functions have attracted widespread attention, the molecular mechanisms by which VTs modulate ROS levels and induce defense responses under abiotic stress conditions are still at a preliminary stage of investigation.

In the face of both climate change and the growing demand for sustainable agriculture, the effective utilization of VTs in enhancing plant stress tolerance and antioxidant regulation has become a prominent research focus. Accordingly, this review summarizes the biosynthetic pathways and regulatory factors involved in VT metabolism and explores how abiotic factors influence their emission dynamics. In addition, the antioxidant and ecological signaling functions of VTs are re-examined within the context of abiotic stress responses. By integrating mechanistic understanding with functional insights, this work aims to provide a theoretical framework for harnessing volatile terpenoids in antioxidant-oriented breeding, reinforcement of plant defense, and the development of natural protective agents.

## 2. Biosynthesis and Key Regulatory Networks of Volatile Terpenoids

### 2.1. Biosynthetic Pathways of Volatile Terpenoids

As the predominant constituents of plant volatile organic compounds (VOCs), VTs constitute the largest and most structurally diverse class, exhibiting a broad range of physiological activities, including antioxidant, antimicrobial, and anti-inflammatory effects [13]. These compounds play pivotal roles in plant growth and development, environmental adaptation, and responses to both biotic and abiotic stressors. Common VTs include monoterpenes, sesquiterpenes, and diterpenes. Structurally, most VTs are biosynthesized from the C_5_ precursors isopentenyl pyrophosphate (IPP) and dimethylallyl pyrophosphate (DMAPP), following the [(C_5_H_8_)_n_] isoprene rule. Hemiterpenes (C_5_H_1__0_), exemplified by photosynthesis-derived isoprene, represent the simplest category. Monoterpenes (C_1__0_H_1__6_), formed via the condensation of one IPP and one DMAPP unit, include conifer-dominant compounds such as α-pinene, limonene, and myrcene [14]. Sesquiterpenes (C_1__5_H_2__4_), synthesized from two IPP units and one DMAPP unit, encompass ecologically significant molecules like β-farnesene and the phytoalexin gossypol. At higher molecular weights, diterpenes (C_2__0_H_3__2_) such as phytol (a chlorophyll-associated alcohol) and gibberellins (key regulators of plant growth) arise from three IPP units and one DMAPP unit. This structural hierarchy—from C_5_ to C_2__0_ frameworks—directly correlates with their functional versatility in plant survival strategies.

**Figure 1:**
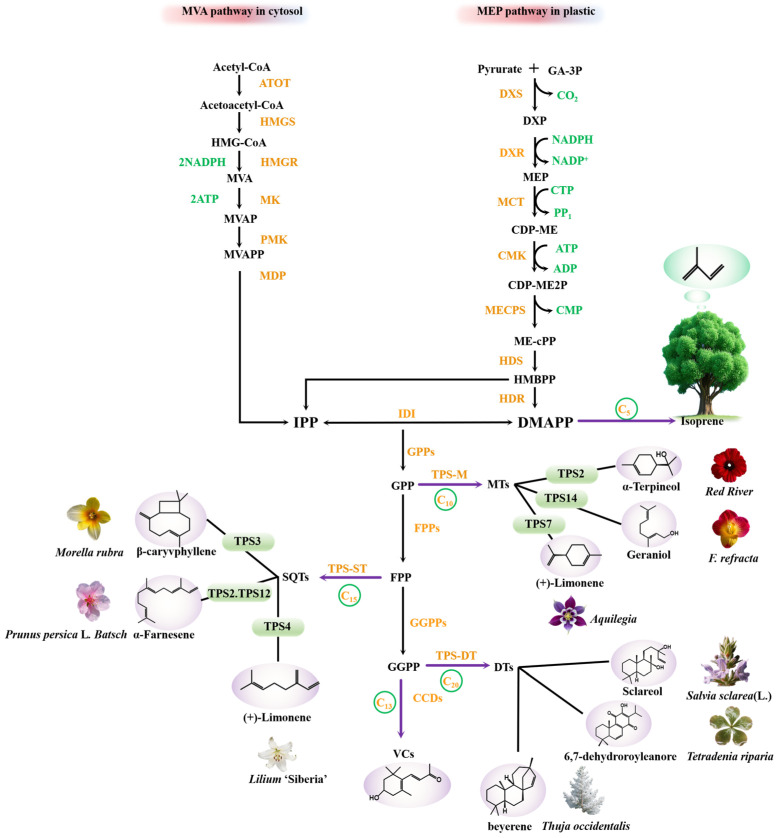
Biosynthesis of plant volatile terpenoids via MVA and MEP pathways catalyzed by distinct terpene synthases. Sources of the specific cases cited in this figure are as follows: *Red River* [23], *F. refracta* [24], *Aquilegia* [25], *Morella rubra* [26], *Prunus persica* L. *Batsch* [27], *Lilium ‘Siberia’* [28], *Salvia sclarea* (L.) [29], *Tetradenia riparia* [30], and *Thuja occidentalis* [31]. The following abbreviations are used in this figure: TPS-M, monoterpene synthase; TPS-ST, sesquiterpene synthase; TPS-DT, diterpene synthase.

While terpenoid biosynthesis involves intricate biochemical networks, it is predominantly governed by two evolutionarily conserved pathways: MVA and MEP pathway (Figure 1).

The MVA pathway, localized in the cytosol [15], facilitates the biosynthesis of terpenoid precursors through a series of enzymatic reactions. The pathway is initiated with acetyl-CoA (acetyl-coenzyme A) serving as the primary substrate. Acetoacetyl-CoA thiolase (ATOT) catalyzes the condensation of two acetyl-CoA molecules to form acetoacetyl-CoA. Hydroxymethylglutaryl-CoA synthase (HMGS) then facilitates the synthesis of HMG-CoA, marking the formation of a key early intermediate. HMG-CoA is subsequently reduced by hydroxymethylglutaryl-CoA reductase (HMGR), yielding mevalonic acid (MVA). Mevalonate kinase (MK) phosphorylates MVA to form mevalonate-5-phosphate (MVAP), which is further phosphorylated by phosphomevalonate kinase (PMK), producing mevalonate-5-diphosphate (MVAPP). Mevalonate diphosphate decarboxylase (MDP) subsequently catalyzes the decarboxylation of MVAPP, yielding IPP, the key C_5_ building block of terpenoid biosynthesis. IPP is isomerized to DMAPP by IPP isomerase (IPI), completing the MVA-derived isoprenoid precursor pool [16,17]. Farnesyl diphosphate synthase (FPPs) catalyzes the head-to-tail condensation of these C_5_ units, leading to the production of farnesyl diphosphate (FPP, C_1__5_)—the central precursor for volatile sesquiterpenes and their derivatives.

The 2-C-methyl-D-erythritol 4-phosphate (MEP) pathway operates exclusively within plastids and executes terpenoid precursor biosynthesis through a conserved enzymatic cascade. The MEP pathway is initiated by the condensation of pyruvate and D-glyceraldehyde-3-phosphate (GA-3P), catalyzed by 1-deoxy-D-xylulose-5-phosphate synthase (DXS), yielding 1-deoxy-D-xylulose-5-phosphate (DXP). In the presence of NADPH, 1-deoxy-D-xylulose 5-phosphate reductoisomerase (DXR) catalyzes the reduction and rearrangement of DXP, resulting in the formation of 2-C-methyl-D-erythritol 4-phosphate (MEP). 2-C-methyl-D-erythritol 4-phosphate cytidylyltransferase (MCT) then transfers a cytidylyl group from cytidine triphosphate (CTP) to MEP, generating 4-(cytidine 5′-diphospho)-2-C-methyl-D-erythritol (CDP-ME). In the presence of ATP, CDP-ME undergoes phosphorylation by 4-diphosphocytidyl-2-C-methyl-D-erythritol kinase (CMK), yielding CDP-ME 2-phosphate (CDP-ME2P). CDP-ME2P is then cyclized by 2-C-methyl-D-erythritol 2,4-cyclodiphosphate synthase (MECPS) to form 2-C-methyl-D-erythritol 2,4-cyclodiphosphate (ME-cPP), accompanied by the release of cytidine monophosphate (CMP). Hydroxy-methylbutenyl diphosphate synthase (HDS) subsequently catalyzes the conversion of ME-cPP into 1-hydroxy-2-methyl-2-butenyl-4-diphosphate (HMBPP). Finally, hydroxy-methylbutenyl diphosphate reductase (HDR) reduces HMBPP, generating the essential isoprenoid precursors, IPP and DMAPP.

The IPP/DMAPP pool is channeled into three prenyltransferase systems: firstly, geranyl diphosphate synthase (GPPs) mediates the head-to-tail condensation of DMAPP with two IPP units to produce geranyl diphosphate (GPP, C10) or its cis-isomer neryl diphosphate (NPP, C10) depending on tissue-specific GPPS isoform expression; secondly, farnesyl diphosphate synthase (FPPs) sequentially adds three IPP units to DMAPP to generate the C15 backbone farnesyl diphosphate (FPP) through precise chain-length control; thirdly, geranylgeranyl diphosphate synthase (GGPPs) extends FPP with additional IPP units to form geranylgeranyl diphosphate (GGPP, C20), which serves as the precursor for diterpenes (C20) via diterpene synthases and carotenoids through phytoene synthase-mediated cyclization [15,18,19,20,21,22]. It should be noted that GPP/NPP and FPP act as precursors for monoterpenes and sesquiterpenes, respectively, in reactions catalyzed by terpene synthases (TPSs) [18]. GGPP is also the precursor for carotenoid formation. Catalyzed by carotenoid cleavage dioxygenases (CCDs), it generates volatile C_1__3_ apocarotenoids.

### 2.2. Regulatory Determinants of Volatile Terpenoid Biosynthesis

#### 2.2.1. Key Enzymes

HMGR, DXS, and DXR serve as key regulatory checkpoints in the upstream metabolism of VTs, governing their biosynthetic cascades through the MVA and MEP pathways.

HMGR is widely recognized as the key rate-limiting enzyme of the MVA pathway, controlling terpenoid flux in the cytosol [32]. HMGR orthologs have been cloned and functionally characterized in diverse plant systems, including *Arabidopsis thaliana* [33], *Salvia miltiorrhiza* [34], *Ginkgo biloba* [35], *Populus trichocarpa* [36], and *Glycine max* [37]. In *Vitis vinifera*, three HMGR paralogs (*VvHMGR1/2/3*) have been identified, among which *VvHMGR3* has been functionally validated to enhance aroma volatile accumulation [38]. Transcriptomic analyses of roses revealed a strong positive correlation between terpene accumulation and the expression levels of *HMGR02* and *HMGR10* [39]. Similarly, targeted suppression of *HMGR* in *Jasminum sambac* significantly reduced α-farnesene emission, confirming its regulatory role in floral volatile biosynthesis [40].

DXR functions as a critical regulatory node in volatile terpenoid biosynthesis, catalyzing the isomerization and NADPH-dependent reduction of 1-deoxy-D-xylulose-5-phosphate (*DOXP*) to generate MEP [41]. This reaction represents the primary rate-limiting step in the DOXP/MEP metabolic pathway [42]. The transcript levels of *DXR* exhibit a strong positive correlation with downstream terpenoid production. In *Arabidopsis thaliana*, genetic upregulation of *DXR* led to a 2.3-fold increase in isoprenoid accumulation [43]. Functional characterization of *LcDXR* from *Litsea cubeba* demonstrated its constitutive expression across roots, stems, leaves, and fruits, with transgenic overexpression significantly enhancing monoterpene biosynthesis. Notably, heterologous expression of *LcDXR* in *Nicotiana benthamiana* resulted in a 5.9-fold increase in key monoterpenes, including limonene, α-pinene, 1,8-cineole, linalool, and terpineol [44], highlighting its conserved regulatory role across plant lineages.

DXS functions as the first committed and rate-limiting enzyme in the MEP pathway. Its functional orthologs have been identified across diverse plant species, including *Arabidopsis thaliana* [45], *Mentha* × *piperita* L. [46], and tomato [47]. Overexpression of *DXS* in *Arabidopsis* resulted in a significant increase in terpene accumulation [48]. Transient overexpression of *AcDXS* in kiwifruit (*Actinidia chinensis*) markedly upregulated the expression of the monoterpene synthase gene *AcTPS1* [49]. In grapevine (*Vitis vinifera* L.), *VvDXS* transcript levels were positively correlated with linalool concentrations and total terpenoid content [50]. In *Ginkgo biloba*, a *DXS* homolog exhibited constitutive expression across vegetative tissues, with *methyl jasmonate* (MeJA)-inducible regulation and a positive correlation with ginkgolide biosynthesis [51]. Collectively, these findings establish *DXS* as a central regulatory node governing terpenoid metabolic flux.

Glycosyltransferases (GTs) comprise a crucial enzyme family that catalyzes the conjugation of free terpenoids into glycosylated, non-volatile derivatives, thereby enabling their intracellular storage [52,53]. In tea (*Camellia sinensis*), terpenoid aroma compounds predominantly exist as glycosidic precursors, with low-temperature exposure inducing a concurrent accumulation of volatile nerolidol and its glycosylated form. Through an integrated analysis of cold-induced gene expression and enzymatic activity screening, UDP-glycosyltransferase *UGT91Q2* was identified as the specific enzyme catalyzing nerolidol glycosylation. *UGT91Q2* exhibits strict substrate specificity toward nerolidol, facilitating its conversion from a free monoterpene to a glycosidically bound form [54].

TPSs, serving as terminal rate-limiting enzymes in downstream terpenoid metabolic pathways, are classified into eight distinct subfamilies, designated TPS-a through TPS-h. Each subfamily plays specialized catalytic roles in regulating the biosynthesis of structurally diverse terpenoids. Specifically, TPS-a primarily governs sesquiterpene biosynthesis, while TPS-b and TPS-g are responsible for cyclic and acyclic monoterpene formation. TPS-c, TPS-e, and TPS-f catalyze the production of GPP, gibberellins, diterpenes, monoterpenes, and sesquiterpenes, with TPS-f also playing a role in gibberellin and diterpene metabolism. Evolutionary divergence has driven functional specialization, wherein TPS-c, TPS-e, and TPS-f are predominantly involved in the biosynthesis of primary metabolites such as gibberellins and abscisic acid, whereas TPS-a, TPS-b, and TPS-g are primarily associated with secondary metabolite biosynthesis [55]. The TPS-h subfamily is mainly found in lycophytes, bryophytes, liverworts, and ferns. The structural diversity of plant terpenoids is attributed to the regulatory roles of TPSs, whose gene expression determines the initiation of metabolic pathways and biosynthesis of associated volatile compounds. For instance, in *Lilium ‘Siberia’*, *LoTPS2* primarily catalyzes the synthesis of (E,E)-α-farnesene [28]. In *Dendrobium officinale*, *DoTPS10* was identified as a key regulatory enzyme controlling linalool biosynthesis [56]. In peach (*Prunus persica*), the transcription factor *PpERF61* activates the expression of *PpTPS1* and *PpTPS3*, thereby promoting the accumulation of the monoterpene geraniol during fruit ripening [57]. Genome-wide analysis of *Perilla frutescens* revealed 109 *PfTPS* genes, with co-expression network analysis across four chemotypes pinpointing critical biosynthetic regulators: *PfTPS46-PL*, *PfTPS46-PK*, *PfTPS18-PA*, and *PfTPS49-PA* encode linalool synthases, whereas *PfTPS47-PA* predominantly produces citronellol, with geraniol as a secondary product [58]. Furthermore, in Newhall sweet orange (*C. sinensis Osbeck*), *CitTPS16* expression modulates E-geraniol synthesis, directly contributing to the formation of fruit flavor-associated aroma compounds [59].

#### 2.2.2. Transcription Factors

Transcription factors (TFs) are sequence-specific DNA-binding proteins that regulate transcriptional initiation by targeting cis-regulatory elements, thereby modulating the expression of structural genes essential for plant developmental processes and specialized metabolic pathways [60,61]. In recent years, multiple TF families have been identified as direct or indirect regulators of VT biosynthetic pathways at the transcriptional level. These transcription factors can coordinately activate gene clusters within specific metabolic modules, significantly enhancing flux efficiency through these pathways. Among the key TF families implicated in VT regulation are AP2/ERF, WRKY, bHLH, bZIP, zinc-finger, MYB, and NAC families, which exert combinatorial control over terpenoid biosynthesis [62,63]. To provide a more systematic overview of the transcriptional regulation of plant terpenoid metabolism, we summarize a set of representative transcription factors that have been functionally validated in different plant species and are involved in the regulation of volatile monoterpene and sesquiterpene biosynthesis (Table 1). Most members of these transcription factor families act as positive regulators, while the MYB and bHLH families exhibit functional diversity, functioning either as activators or repressors depending on their target genes and expression contexts. Notably, some transcription factors show organ- or species-specific expression patterns, further reflecting the spatiotemporal complexity of terpenoid metabolic regulation. Overall, the transcriptional control of volatile terpenoid biosynthesis in plants is highly complex and modular, and many potential regulators and their underlying mechanisms remain to be fully elucidated.

MYB TFs, one of the largest transcription factor families in plants [64], predominantly regulate VT biosynthesis by modulating the transcription of core structural genes. In sweet osmanthus (*Osmanthus fragrans*), functional characterization of *OfMYB1R114* and *OfMYB1R70* revealed their roles in promoting β-ionone biosynthesis, whereas *OfMYB1R201* functions as a repressor, inhibiting β-ionone production [65]. In tomato (*Solanum lycopersicum*), *SlMYB75* enhances anthocyanin accumulation and potentiates the biosynthesis of volatile aroma compounds in fruits. Overexpression of *SlMYB75* activates the promoters of lipoxygenase C (*LOXC*), aromatic amino acid decarboxylase 2 (*AADC2*), and *TPS* genes, thereby boosting the production of diverse aromatic volatiles, including aldehydes, phenylpropanoid derivatives, and terpenoid volatiles [66]. A dual regulatory mechanism governing glandular trichome development and diterpenoid biosynthesis was elucidated in *Conyza blinii*. Transient overexpression or RNA interference (RNAi)-mediated silencing of *CbMYB108* in leaves respectively increased or decreased diterpenoid accumulation and peltate glandular trichome density. Mechanistically, *CbMYB108* upregulates the expression of 1-deoxy-D-xylulose 5-phosphate synthase (*CbDXS*) and geranylgeranyl diphosphate synthase (*CbGGPPS*) in the diterpenoid biosynthetic pathway [67]. In *Lilium ‘Siberia’*, transient silencing of *LiMYB305* in petals significantly downregulated the expression of monoterpene biosynthesis-related genes *LiOcS* and *LiMyS*, leading to a marked reduction in the emissions of linalool, ocimene, and myrcene. This functional analysis unequivocally establishes *LiMYB305* as a master transcriptional regulator of monoterpene biosynthetic pathways [68].

MYC TFs, belonging to the bHLH family, have been identified and functionally characterized in multiple plant species, including *Arabidopsis thaliana* [69,70], *Solanum lycopersicum* [71], *Artemisia annua* [72], and *Camellia sinensis* [73], where they primarily regulate monoterpene and sesquiterpene biosynthesis. In wintersweet (*Chimonanthus praecox* L.), *CpMYC2* and *CpbHLH13* function as transcriptional activators, positively regulating the biosynthesis of linalool and β-caryophyllene by upregulating their biosynthetic gene clusters [74]. Transgenic overexpression of *LaMYC7* in lavender (*Lavandula angustifolia*) significantly increased linalool and caryophyllene accumulation [75]. Similarly, overexpression of *AsMYC2* in agarwood (*Aquilaria sinensis*) upregulated *TPS11* and *TPS21* expression, leading to enhanced sesquiterpene production through coordinated pathway activation [76]. These findings collectively establish MYC TFs as evolutionarily conserved regulators of terpenoid diversification.

WRKY TFs orchestrate the production of all major classes of secondary metabolites—including terpenes, phenylpropanoids, and alkaloids—by modulating the expression of genes encoding enzymes within their respective biosynthetic pathways [77,78]. CrWRKY1 functions as a positive regulator of terpenoid indole alkaloid (TIA) biosynthesis in *Catharanthus roseus* [79]. *GaWRKY1*, a WRKY transcription factor, activates CAD1-A expression by binding to the W-box cis-element within its promoter, thereby regulating sesquiterpenoid biosynthesis in cotton (*Gossypium arboreum*) [80]. 

TF genes assemble into protein complexes that coordinately control the expression of enzymatic genes within terpenoid biosynthetic pathways [79,81]. In freesia (*Freesia hybrida*), the FhMYC2 protein interacts with FhMYB21Ls to form a transcriptional repressor complex. This complex suppresses the expression of *FhTPS1* by competitively inhibiting *FhMYB21Ls* from binding to the *FhTPS1* promoter during floral development [82]. A jasmonate (JA)-responsive regulatory cascade was identified in *Curcuma wenyujin*, where *CwJAZ4/9* proteins physically interact with *CwMYC2* to negatively modulate JA-induced terpenoid biosynthesis (e.g., curcumol, zederene and elemene). Transgenic overexpression of *CwJAZ4/9* significantly reduced terpenoid accumulation in root hairs, while RNAi-mediated silencing enhanced JA-dependent terpenoid production, confirming their antagonistic regulatory roles [83].

#### 2.2.3. Plant Hormones

Plant hormones coordinate the dynamic reprogramming of secondary metabolism during development, defense priming, and environmental adaptation via complex signaling networks. Key phytohormones such as jasmonic acid (JA), salicylic acid (SA), ethylene (ET), and abscisic acid (ABA) activate specific TFs to regulate the expression of TPS genes, thereby modulating the biosynthesis and emission of VTs, including monoterpenes, sesquiterpenes, and diterpenes. This hormonal integration ensures precise spatiotemporal regulation of floral scent emission and stress-induced volatile signaling, which are critical for plant ecological fitness.

JA plays a central role in regulating terpenoid biosynthesis by integrating into both the MVA and MEP pathways. JA directly modulates the expression of key biosynthetic genes, including *DXS*, *DXR*, *PMK*, and *TPS*, while indirectly coordinating TFs such as the MYB, bHLH, and WRKY families to fine-tune terpenoid production [84]. The JA signaling module consists of the F-box protein COI1, JAZ transcriptional repressors, and the central transcription factor MYC2. Upon JA accumulation, COI1, together with bioactive jasmonoyl-isoleucine (JA-Ile), facilitates the assembly of the SCF^COI1 E3 ubiquitin ligase complex, which specifically interacts with JAZ repressors. This interaction triggers JAZ ubiquitination and subsequent proteasomal degradation, thereby releasing MYC2 [85,86] to promote the biosynthesis of terpenoid metabolites. Functional studies in *Fragaria × ananassa* show that JA treatment induces the expression of *FaTPS1*, which catalyzes the synthesis of germacrene D, with *FaMYC2* acting as the JA-responsive TF driving this regulatory cascade [87]. Similarly, exogenous methyl jasmonate (MeJA) application in *Solanum lycopersicum* upregulates the terpene synthase gene *SlJIG*, enhancing sesquiterpene biosynthesis and conferring insect resistance through volatile-mediated defense mechanisms, thereby illustrating the evolutionary conservation of JA-mediated terpenoid regulation across plant species [88].

ET, a gaseous phytohormone, regulates plant growth, developmental transitions, and responses to environmental stress through intricate signaling networks [89]. In grapevine (*Vitis vinifera*), the ET-responsive transcription factor *VviERF003* upregulates *VviGT14*, enhancing the biosynthesis of glycosylated monoterpenes [90]. Notably, ET acts upstream of JA and SA signaling while functioning downstream of nitric oxide (NO) and hydrogen peroxide (H_2_O_2_) pathways, serving as a key mediator in endophytic fungus-induced sesquiterpenoid production in *Atractylodes lancea* [91].

**Table 1:** Involvement of transcription factors in the biosynthesis of plant volatile terpenoids.

Transcription Factor	Gene Name	Species Name	Regulatory Substance	Reference
MYB	*MsMYB*	*Mentha spicata*	Inhibits limonene and carvone biosynthesis	[92]
	*AlMYB59*	*Atractylodes lancea*	Promotes β-eudesmol, atractylon, and atractylone biosynthesis	[93]
	*FhMYB21L1*, *FhMYB21L2*	*Freesia hybrida*	Involved in monoterpene and sesquiterpene biosynthesis	[82]
	*LiMYB108*	*Lilium*	Promotes ocimene and linalool biosynthesis	[94]
	*AmMYB24*	*Antirrhinum majus*	Promotes ocimene biosynthesis	[95]
	*JsMYB108*, *JsMYB305*	*Jasminum sambac*	Involved in monoterpene and sesquiterpene biosynthesis	[96]
	*MYB*	*Saussurea lappa*	Involved in sesquiterpene lactone biosynthesis	[97]
	*HcMYB1*, *HcMYB2*	*Hedychium coronarium*	Promotes linalool biosynthesis	[98]
	*SlMYB75*	*Solanum lycopersicum* L.	Promotes terpene volatile biosynthesis	[66]
	*HcMYB*	*Hedychium coronarium*	Promotes terpene biosynthesis	[99]
	*OfMYB1R114*, *70*, *201*	*Osmanthus fragrans*	Promotes β-ionone biosynthesis	[65]
	*CsMYB68*, *147*, *148*, *193*	*Camellia sinensis*	Promotes monoterpene and sesquiterpene biosynthesis	[100]
	*MYB24*	*Vitis vinifera* cv. *‘Béquignol’*	Involved in monoterpene biosynthesis	[101]
	*MYB5*	*Rosa rugosa*	Involved in sesquiterpene biosynthesis	[102]
bHLH	*MYC2*	*Arabidopsis thaliana*	Promotes sesquiterpene biosynthesis	[69]
	*PbbHLH4*	*Phalaenopsis orchids*	Promotes monoterpene biosynthesis	[103]
	*CpMYC2*, *CpbHLH13*	*Chimonanthus praecox* L.	Promotes β-caryophyllene and linalool biosynthesis	[74]
	*bHLH35*	*Osmanthus fragrans*	Promotes linalool and linalool oxide biosynthesis	[104]
	*AabHLH2*, *3*	*Artemisia annua* L.	Promotes sesquiterpene lactone biosynthesis	[105]
	*LibHLH22*, *63*	*Lilium ‘Siberia’*	Promotes linalool and ocimene biosynthesis	[106]
	*SlMYC1*	*Solanum lycopersicum*	Promotes monoterpene biosynthesis in leaves while inhibiting sesquiterpene biosynthesis in stem trichomes	[107]
	*PpbHLH1*	*Prunus persica* L.	Promotes linalool biosynthesis	[108]
	*LaMYC4*	*L. angustifolia*	Promotes volatile organic compound biosynthesis	[109]
AP2/ERF	*MdERF3*	*Malus domestica*	Promotes α-farnesene biosynthesis	[110]
	*CitERF71*	*Citrus sinensis Osbeck*	Promotes E-geraniol biosynthesis	[59]
	*PpERF5,7*	*Prunus persica*	Promotes linalool biosynthesis	[111]
WRKY	*AaWRKY40*	*Artemisia annua*	Promotes terpene biosynthesis	[112]
	*CrWRKY1*	*Catharanthus roseus*	Promotes terpene indole alkaloid biosynthesis	[79]
	*OfWRKY139*	*Sweet Osmanthus*	Promotes monoterpene biosynthesis	[113]
NAC	*AaNAC2*, *3*, *4*	*Actinidia chinensis Planch*	Promotes monoterpene biosynthesis	[114]
	*GoNAC42*	*Gossypium hirsutum*	Promotes monoterpene biosynthesis	[115]
	*NAC-NOR*	*Solanum lycopersicum*	Promotes volatile organic compound biosynthesis	[116]

## 3. Regulatory Mechanisms of Volatile Terpenoids in Abiotic Stress Responses

### 3.1. Protective Roles and Mechanisms of Volatile Terpenoids Under Abiotic Stress

Under abiotic stresses such as drought, high temperatures, and ozone exposure, plants rapidly initiate early signaling cascades involving the burst of ROS and transient calcium fluxes (Ca^2^^+^). ROS are primarily generated by photosystem II electron leakage in chloroplasts, disruptions in mitochondrial electron transport chains, and peroxisomal β-oxidation [117,118]. The excessive accumulation of ROS—particularly superoxide anion (O_2_^−^), hydrogen peroxide (H_2_O_2_), and hydroxyl radicals (·OH) [119]—results in oxidative damage, including lipid peroxidation, protein denaturation, and ultimately, programmed cell death (PCD) [117]. In parallel, stress signals activate calcium channels on plasma membranes and organelles, triggering Ca^2^^+^ release from extracellular reservoirs or intracellular stores such as the vacuole and endoplasmic reticulum, leading to a ‘calcium burst.’ Elevated cytosolic Ca^2^^+^ binds to calmodulin (CaM) and calcium-dependent protein kinases (CDPKs), propagating the signal to downstream modules, including mitogen-activated protein kinase (MAPK) cascades, hormonal pathways (e.g., JA, ABA, SA, and ET), and ROS-producing or scavenging systems. These interactions ultimately activate stress-responsive gene expression. Moreover, a positive feedback loop exists between ROS and Ca^2^^+^: ROS promote Ca^2^^+^ influx, while Ca^2^^+^ in turn stimulates NADPH oxidases to generate more ROS, forming a self-amplifying signal network that rapidly escalates and disseminates stress cues throughout plant tissues [120,121,122].

**Figure 2:**
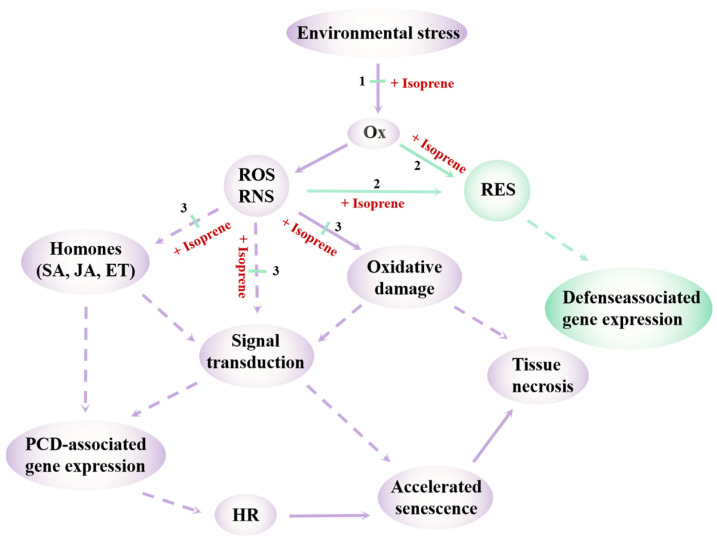
The ‘single biochemical mechanism for multiple physiological stressors’ model. The model shows how oxidative damage resulting from environmental stress occurs (in purple) and how volatile isoprenoids may exert protective effects through antioxidant activity (in green). Solid lines represent direct reactions, and broken lines represent indirect reactions. Environmental stress (high light, temperature, and ozone exposure) causes oxidative stress (Ox), which results in the production of ROS (for example, hydrogen peroxide, singlet oxygen, and superoxide) and reactive nitrogen species (RNS; for example, nitric oxide, peroxynitrite). These compounds initiate cell signaling directly and also through interactions with the hormonal response network, as well as causing further direct oxidative damage. Different stresses trigger different response pathways. For example, ozone exposure also triggers a response that overlaps with biotic stress responses through the plant hormone network; salicylic acid (SA), jasmonic acid (JA), and ethylene (ET) trigger signal cascades that initiate programmed cell death (PCD), resulting in accelerated senescence via an inappropriate hypersensitive response (HR). Isoprene may act at several different levels to arrest oxidative stress-response processes. (1) Because it is lipophilic, isoprene may physically stabilize hydrophobic interactions in membranes, minimizing lipid peroxidation and reducing oxidative stress and downstream buildup of ROS/RNS. (2) Isoprene may react with ROS/RNS to produce reactive electrophile species (RES) such as methacrolein and methylvinylketone (products of isoprene/ozone reaction), which are known to induce antioxidant and other defenses. If the stressor is itself an ROS (for example, ozone), isoprene may react directly with the stressor. (3) Direct antioxidant behavior (scavenging ROS/RNS) also prevents accumulation to damaging levels, thus preventing further oxidative damage. As a consequence, ROS/RNS-activated signal cascades and PCD pathways that normally result in tissue necrosis are prevented.

To mitigate oxidative damage, plants must promptly activate antioxidant defenses to maintain redox homeostasis. In addition to enzymatic antioxidants such as superoxide dismutase (SOD), catalase (CAT), and ascorbate peroxidase (APX), and non-enzymatic antioxidants like glutathione (GSH) and Ascorbic acid (AsA) [123,124,125], increasing attention has been given to volatile terpenoids as small-molecule antioxidants that confer protection at both the cellular and systemic levels [126,127]. Antioxidants function by directly neutralizing ROS or interrupting free radical chain reactions, thereby preventing oxidative damage [128]. Isoprene, a volatile hemiterpenoid, has emerged as a multifunctional antioxidant [129,130,131]. Its conjugated double bonds enable direct scavenging of ·OH, forming 2-methyltetrols in aqueous solution [132]. Given the high reactivity and cellular toxicity of ·OH, isoprene’s neutralization capacity significantly reduces membrane lipid peroxidation and limits oxidative injury. Under high light stress, isoprene also quenches singlet oxygen (^1^O_2_) generated at the thylakoid membrane, thereby maintaining photosynthetic efficiency and preserving membrane integrity [133]. Additionally, isoprene reacts with ozone in the leaf boundary layer, scavenging intercellular ozone, reducing O_3_ concentration in mesophyll tissues, and preventing the formation of reactive nitrogen species [134], thereby protecting plant tissues from oxidative damage. Under O_3_ stress, the inhibition of isoprene biosynthesis by fosmidomycin leads to marked accumulation of H_2_O_2_, elevated lipid peroxidation, and enhanced activity of antioxidant enzymes in plant leaves. Exogenous fumigation with isoprene partially reverses these effects, restoring H_2_O_2_ levels and lipid peroxidation to values closer to those observed in non-inhibited control leaves [134]. Furthermore, isoprene-emitting plants show higher concentrations of reduced AsA compared to non-emitting counterparts, indicating a reduced reliance on the enzymatic antioxidant system [135]. Collectively, these findings support the role of isoprene as a key volatile antioxidant that mitigates oxidative burden and contributes to cellular homeostasis under thermal stress. Notably, isoprene oxidation products such as methyl vinyl ketone (MVK) and methacrolein (MACR) [136], known as reactive electrophilic species (RES) [137,138], can induce antioxidant gene expression and activate systemic signaling pathways mediated by SA, JA, and ET [136,138]. Thus, isoprene performs a dual function: directly scavenging ROS and indirectly amplifying stress defense via signal transduction. Based on these findings, Vickers et al. proposed a ‘common biochemical mechanism’ hypothesis [139] (Figure 2), suggesting that oxidative damage represents a shared core across diverse abiotic stresses and that ROS-targeted mitigation is a unifying protective strategy. This concept provides a comprehensive framework for understanding oxidative regulation in plant stress biology.

In the complex defense system that plants deploy against abiotic stressors, volatile terpenoids beyond isoprene—particularly monoterpenes (C_1__0_)—have demonstrated significant antioxidant capacity. These compounds contribute to multi-dimensional protection by scavenging ROS, stabilizing membrane structures, modulating signal transduction pathways, and inducing the expression of antioxidant-related genes. For instance, α-pinene and β-pinene have been shown to enhance the thermal stability of the photosynthetic electron transport chain under heat stress, exhibiting functions comparable to isoprene in thermotolerance [140,141]. Additionally, monoterpenes can directly react with ozone in the leaf boundary layer [137], thereby reducing O_3_ penetration into mesophyll tissues and alleviating oxidative injury. Structurally, monoterpenes are composed of two isoprene units and can form acyclic, monocyclic, or bicyclic skeletons, often bearing functional groups such as alcohols, ketones, and aldehydes. This chemical diversity underlies their broad reactivity and biological functionality [142]. Studies in *Q. ilex* L. [143] and thyme [144] have shown that monoterpenes like linalool, geraniol, and α-pinene are released in large quantities under drought or heat conditions [145]. In cork [146] oak (*Quercus suber*), fumigation with α-pinene significantly reduced H_2_O_2_ and malondialdehyde (MDA) accumulation, enhanced APX activity, and delayed photosynthetic decline under heat stress. Beyond biochemical antioxidant roles, monoterpenes also participate in the integration of stress signaling. Under cold stress, expression of monoterpene biosynthetic genes in rice is upregulated and co-activated with the MAPK cascade and ethylene signaling pathways, indicating monoterpenes as signaling intermediates [147]. Under drought conditions, monoterpene accumulation strongly correlates with antioxidant enzyme activity, suggesting a dual role as non-enzymatic ROS scavengers and membrane protectants [148]. In wild rose petals, a negative correlation between geraniol levels and H_2_O_2_ content has been observed, supporting the idea that monoterpene-rich organs maintain lower oxidative stress, potentially delaying senescence and extending floral longevity [149].

Compared to monoterpenes, sesquiterpenes (C_1__5_)—such as β-caryophyllene and farnesene—are more hydrophobic and thus more likely to integrate into lipid membranes. This property enhances their capacity to protect membranes from lipid peroxidation, particularly under high-temperature or salinity stress [150,151,152]. In addition to directly neutralizing ROS as lipid-soluble radical scavengers, sesquiterpenes have been reported to induce the expression of heat shock proteins (HSPs) and modulate osmoprotectant accumulation, thereby supporting plant tolerance under compound stress environments [139,153]. For example, in Iranian basil, both linalool (a monoterpene) and γ-cadinene (a sesquiterpene) showed significant increases in response to moderate drought stress, reflecting inducible metabolic reprogramming [154]. In *S. dolomitica*, studies under varying stress conditions reveal divergent metabolic responses: under warming, terpenoid emission was reduced while levels of ascorbate and APX activity increased, suggesting a compensatory reliance on non-volatile antioxidants; in contrast, during drought stress, terpenoid release was maintained, indicating a carbon-based defense strategy. This pattern suggests that terpenoid-mediated antioxidative responses are highly plastic and tailored to specific stress types [155]. Furthermore, (E)-β-farnesene has been observed to rapidly react with ozone in experimental chambers, significantly lowering O_3_ concentrations and mitigating ozone-induced oxidative damage. This positions certain sesquiterpenes not only as cellular antioxidants but also as environmental oxidative shields. Collectively, these findings demonstrate that both monoterpenes and sesquiterpenes are indispensable components of plant antioxidant defenses under abiotic stress [156]. Their structural diversity, regulated storage and emission patterns, and integration with stress signaling networks confer them with multifunctional roles in maintaining redox homeostasis, stabilizing photosynthetic machinery, and enhancing overall plant resilience.

**Figure 3:**
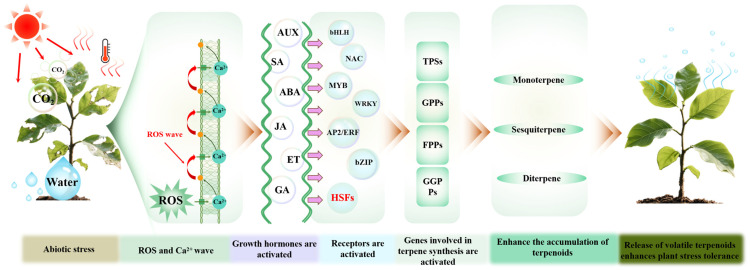
The signaling cascade of volatile terpenoid biosynthesis and their role in enhancing plant resilience to abiotic stress. The figure illustrates the signaling cascade triggered by abiotic stress in plants, where the accumulation of reactive oxygen species (ROS) generates a ROS wave, which activates calcium (Ca^2^^+^) flux and initiates early signaling events. These signals subsequently activate a series of plant growth hormones, including auxin (AUX), salicylic acid (SA), abscisic acid (ABA), jasmonic acid (JA), ethylene (ET), and gibberellins (GA). Different hormone signaling pathways then activate corresponding transcription factors (such as MYB, WRKY, and bZIP) and heat shock proteins (HSFs). For instance, the auxin signaling pathway activates ARF, the ABA pathway activates bZIP and ABF, the ethylene pathway activates ERF, and the JA and SA pathways activate NAC. Finally, these transcription factors upregulate genes involved in terpenoid biosynthesis, including geranyl pyrophosphate synthase (GPPs), farnesyl pyrophosphate synthase (FPPS), geranylgeranyl pyrophosphate synthase (GGPPS), and terpene synthases (TPSs), leading to enhanced accumulation and release of terpenoids, which in turn improve the plant’s resilience to abiotic stresses.

Abiotic stress is one of the major external factors influencing the emission of plant volatile compounds. We provide a concise overview of the regulatory cascade governing volatile terpenoid emission in plants under abiotic stress conditions (Figure 3). Upon exposure to environmental stimuli, plants rapidly accumulate ROS and Ca^2^^+^ signaling, which serve as early signals [157]. These primary signals subsequently activate downstream phytohormone pathways. The hormone signals are further integrated into specific TFs, which, once activated, bind to the promoter regions of key structural genes involved in volatile terpenoid biosynthesis [158,159,160]. Ultimately, the coordinated expression of these biosynthetic genes promotes the synthesis and emission of volatile terpenoids, playing vital roles in plant stress adaptation and ecological communication [161,162].

### 3.2. Temperature Stress

As a key environmental regulator, temperature imposes multifaceted effects on plant physiological metabolism and the emission dynamics of VTs. Studies have demonstrated that temperature fluctuations can directly influence both the emission rates and compositional profiles of plant VOCs. Under elevated temperature stress, plants typically increase terpene emissions as an adaptive response [163,164], with isoprene and monoterpenes being the dominant volatile fractions, whereas sesquiterpenes and diterpenes contribute comparatively less. This temperature-dependent emission pattern results from a complex interplay of physiological processes, including stomatal regulation, photosynthetic electron transport efficiency, and the thermal sensitivity of key enzymatic activities [139]. Mechanistically, isoprene biosynthesis occurs via the MEP pathway within chloroplasts, utilizing photosynthetic intermediates as substrates [165]. The rate-limiting step in DMAPP biosynthesis, along with the catalytic efficiency of isoprene synthase (IspS), exhibits pronounced temperature sensitivity, making isoprene emissions particularly responsive to thermal fluctuations [166]. For instance, the heterologous expression of *IspS* in *Arabidopsis thaliana* enables non-isoprene-emitting plants to produce temperature-dependent isoprene emissions, with significant increases under thermal stress [167]. This emission pattern closely aligns with the temperature-dependent activity profiles of IspS enzymes observed in *Populus tremula* × *Populus tremuloides* [168]. However, temperature effects exhibit threshold characteristics: *Pueraria lobata* leaves grown under a constant 19 °C (day/night) fail to emit isoprene, whereas isoprene biosynthesis becomes inducible above 20 °C [169]. Notably, studies on temperature responses in *Quercus robur* and *Populus deltoides* reveal biphasic emission patterns—isoprene emissions increase with temperature up to 35–40 °C, beyond which they decline. This suppression correlates with heat-induced metabolic constraints, including depletion of DMAPP and subsequent limitation of IspS activity [170,171]. Collectively, these findings underscore the pivotal role of enzymatic thermostability in regulating temperature-dependent terpenoid emissions.

Regarding the protective functions of isoprene under high temperatures, existing studies propose multiple complementary mechanisms. In 1995, Sharkey first proposed membrane stabilization as a mechanistic explanation for the function of isoprene. Due to its lipophilic nature and localization within chloroplasts, isoprene is likely partitioned into the lipid phase of thylakoid membranes. When heat stress occurs, increased fluidity of thylakoid membranes reduces the efficiency of photosynthesis. Isoprene enhances the hydrophobicity of membranes, thereby stabilizing the thylakoid membranes and enabling plants to exhibit improved thermotolerance [172,173]. Isoprene prevents alterations in the PSII microenvironment [174], ensuring a more stable and uniform distribution of light-harvesting complexes while maintaining thylakoid membrane rigidity under elevated temperatures to support photosynthetic efficiency. Second, isoprene acts as an antioxidant, protecting plants from oxidative stress by quenching ROS [130,134], thereby enhancing resistance to abiotic stressors. Additionally, some studies suggest that isoprene mitigates heat stress by improving photosynthetic stability.

Temperature regulates terpenoid metabolism not only during plant growth but also significantly impacts postharvest fruit physiology. In strawberry (*Fragaria × ananassa*), cold treatment (4 °C), ambient storage (25 °C), and heat treatment (37 °C) differentially regulate aroma biosynthesis, with *FaNES* and other volatile synthesis genes reaching peak expression under heat stress. Both cold and heat treatments decrease geraniol proportions relative to ambient conditions, while HSFs enhance aroma compound production in response to thermal fluctuations [175], indicating temperature-dependent differential regulation of metabolic pathways via HSFs. During postharvest ripening, trans-nerolidol levels increase, with dark incubation at 15 °C facilitating its accumulation via *FaNES1* activation, whereas β-linalool remains unaffected by light or temperature changes. Notably, cultivar-specific temperature responses show contrasting trends: after 9 days of cold storage, *‘Akihime’* exhibits higher terpenoid content than under room temperature storage, whereas ‘Sweet Charlie’ accumulates fewer terpenoids at 15 °C than at 25 °C [176].

In summary, temperature plays a complex and multifaceted role in regulating plant volatile terpenoid emissions, with substantial interspecies differences in thermal responsiveness. With escalating global warming, plant physiological states are undergoing significant alterations, highlighting the urgent need to investigate temperature-driven modulation of terpenoid metabolism during key developmental stages as a research priority.

### 3.3. CO_2_ Concentration

At the leaf level, elevated CO_2_ concentrations typically suppress isoprene emissions in both controlled environments [177,178] and open ecosystems [179]. This suppression is primarily attributed to CO_2_-enhanced photosynthesis, which redirects key photosynthetic precursors, such as phosphoenolpyruvate (PEP), toward CO_2_ fixation, thereby reducing the availability of DMAPP—the direct precursor of isoprene—for *IspS*-mediated biosynthesis [177]. Notably, Pegoraro et al. [178] reported that isoprene emissions remained suppressed even after plants were re-exposed to ambient CO_2_ levels, indicating potential long-term metabolic reprogramming. Further mechanistic studies by Calfapietra et al. [180] on *Populus tremuloides* clones with varying isoprene sensitivity demonstrated a linear decline in emissions with increasing intracellular CO_2_, linking clonal differences to variations in *IspS* gene expression and enzymatic activity rather than DMAPP substrate availability. In *Quercus rubra* L., isoprene emissions increased with photon flux density (PFD) under low CO_2_ conditions (≤200 μmol·mol^−1^) but sharply declined beyond this threshold, suggesting that CO_2_ modulates ATP allocation and carbon metabolism within photosynthetic partitioning [181]. Untargeted metabolomics analyses conducted by Danielle A. Way et al. [182] on wild-type and RNAi-suppressed *IspS* poplars under elevated CO_2_ revealed metabolic compensation mechanisms. Specifically, the suppression of isoprene biosynthesis was associated with increased antioxidant levels, alterations in lipid composition, and enhanced accumulation of photosynthetic pigments. Notably, this CO_2_-driven metabolic shift contrasts with the heat-induced upregulation of isoprene, underscoring the context-dependent protective functions of isoprene, which likely arise from the integrated regulation of multiple stress-response pathways through combinatorial defense signaling networks.

Elevated CO_2_ concentrations elicit divergent responses in the emission of BVOCs, particularly monoterpenes, across different plant species. Staudt et al. [183] reported a 1.8-fold increase in monoterpene emissions from *Quercus ilex* under elevated CO_2_, potentially due to a transient carbon surplus resulting from CO_2_-enhanced biomass productivity, which redirected metabolic flux toward the synthesis of secondary metabolites. Conversely, Loreto et al. [184] observed significant reductions in monoterpene emissions from *Q. ilex* grown at 700 ppm CO_2_, which were attributed to suppressed monoterpene synthase enzymatic activity. In contrast, studies on conifers (*Pinus* spp. and *Picea* spp.) revealed no significant CO_2_-driven changes in foliar monoterpene concentrations or emissions [185,186], likely due to the metabolic costs associated with the development and maintenance of resin ducts—specialized secretory structures for monoterpene storage—rather than the biosynthesis of monoterpenes themselves [187]. This variability has been attributed to species-specific carbon allocation strategies between growth (primary metabolism) and defense (secondary metabolism). Despite significant progress, the regulatory mechanisms underlying CO_2_–monoterpene interactions remain incompletely understood. The current literature reveals significant inconsistencies regarding the effects of CO_2_ on BVOC emissions in woody plants and crops, with no consensus on the direction of these responses. Critical knowledge gaps remain in understanding the long-term acclimation dynamics and ecological consequences of CO_2_-modulated BVOC fluxes, highlighting the need for integrated molecular–physiological studies to unravel this complexity.

### 3.4. Light

Light intensity, a critical environmental factor influencing plant growth and metabolism, exerts significant regulatory effects on VT emissions, especially the light-dependent release of isoprene and monoterpenes. Studies demonstrate that emission rates of isoprene and monoterpenes exhibit a light-dependent increase with rising photosynthetically active radiation (PAR) until reaching species-specific saturation thresholds, where the light saturation points (LSPs) for isoprene and monoterpenes serve as key determinants of characterizing this photoresponse [188]. Specifically, the emission rate of isoprene increases gradually between 0 and the saturation point of PAR, as exemplified by *Loropetalum chinense* and *Nandina domestica*, which reach maximum isoprene emission rates of 3279.21 and 7355.17 pmol m^−2^ s^−1^ at 1000 μmol m^−2^ s^−1^ PAR [189]. Similarly, *Mangifera indica* shows an increase in isoprene emissions from 6 to 16 nmol m^−2^ s^−1^ as PAR rises from 500 to 1000 μmol m^−2^ s^−1^ [190], highlighting isoprene high photosensitivity. Monoterpene emissions demonstrate stronger PAR responsiveness than isoprene, with greater variability in light saturation thresholds and unstable post-LSP emission patterns [188]. For example, European beech (*Fagus sylvatica*) shows minimal monoterpene emission fluctuations under varying PAR, while early- and late-flowering Norway spruce (*Picea abies*) achieve monoterpene emission saturation at 500 and 1000 μmol m^−2^ s^−1^ PAR, respectively [191], highlighting interspecies divergence in photoresponsiveness. Staudt et al. [192] classified BVOC emissions into light-dependent (LD) and non-light-dependent (NLD) types based on enzymatic versus storage-pool dynamics in Aleppo pine (*Pinus halepensis*). LD emissions (e.g., (E)-β-ocimene and linalool) follow enzymatic kinetics, while NLD emissions (e.g., α-pinene and myrcene) originate from storage pools, showing temperature-dependent exponential increases without light regulation. Conversely, all rosemary (*Rosmarinus officinalis*) VOCs, including oxygenated derivatives, originate from storage pools, suggesting anatomical and physiological constraints on emission mechanisms.

Light perception through photoreceptors (phytochromes, cryptochromes, and UVR8) enables spectral quality-dependent regulation of terpenoid biosynthesis [193]. Illumination intensifies photosynthetic activity in plants and concurrently influences the pool size of DMADP, a precursor for isoprene biosynthesis in leaves, thereby increasing the rate of isoprene production [194,195]. Certainly, Ca^2^^+^ signaling contributes to the biosynthesis and emission of monoterpenes regulated by light intensity. The increase in light intensity initially triggers Ca^2^^+^ influx into the cytoplasm, followed by the activation of downstream monoterpene synthase gene expression, which in turn governs the biosynthesis and emission of monoterpenes [196]. In strawberry, red light upregulates *FaNES1* expression, enhancing trans-nerolidol synthesis, while blue light reduces terpenoid accumulation, and green/violet light suppresses *FaNES1* activity and emission rates [197]. High light/heat stress shifts BVOC profiles: oxygenated monoterpenes and sesquiterpenes increase, while monoterpene hydrocarbon emissions decline, concomitant with photosynthetic inhibition (reduced electron transport rates, ETRs) and precursor limitation [198]. Sun et al. [199] demonstrated that monoterpenes and other BVOCs played a dominant role in stress resistance enhancement in *Hybrid poplars* under severe photoinhibitory conditions, whereas isoprene exhibited diminished protective efficacy under such metabolic bottleneck.

### 3.5. Water

Water plays a crucial role in plant physiology as both a metabolic solvent and a regulator of growth. Moderate drought stress enhances mechanisms that protect against oxidative stress, thereby modulating terpenoid biosynthesis and emission. As the primary sites of CO_2_ exchange, stomata exhibit reduced conductance under elevated CO_2_ conditions, primarily due to acidification-induced guard cell closure. This response functions as an adaptive mechanism to enhance water-use efficiency by maintaining photosynthetic rates while minimizing transpiration [200,201,202]. Drought effects on net photosynthesis (A_n_ₑₜ) and BVOC emission rates follow a biphasic pattern: BVOC emissions initially increase, then decline with prolonged drought severity [169]. Parveen et al. [203] observed a ~50% increase in isoprene emissions in *Ficus septica* after 4 days of drought, despite a ~70% reduction in A_n_ₑₜ and stomatal conductance (gₛ), while Pegoraro et al. documented a 3% decline in isoprene emissions in *Quercus virginiana* after 6 days of drought (with a 65.4% reduction in A_n_ₑₜ and 72.9% in gₛ), highlighting species-specific responses. Exogenous application of monoterpenes to tomato leaves has been shown to alleviate drought-induced oxidative stress primarily by directly quenching reactive species and/or enhancing endogenous antioxidant responses. Such treatments significantly reduced the accumulation of H_2_O_2_ and mitigated lipid peroxidation, as indicated by decreased MDA levels. Notably, treatment with low concentrations of monoterpenes (1.25 mM) proved most effective in relieving oxidative stress, whereas higher concentrations (5 mM) showed intermediate efficacy compared to both the low-dose and control treatments. Furthermore, high-dose monoterpene application markedly increased the activities of antioxidant enzymes, including SOD and APX. However, the exogenous application of monoterpenes did not improve photosynthetic performance or prevent the decline in photosynthetic efficiency. These findings suggest that monoterpenes primarily function as oxidative protectants under water deficit by strengthening the antioxidant defense system while offering limited protection to the photosynthetic apparatus [204].

Reactive terpenoids like α-pinene, β-pinene [205], and α-farnesene [206] show elevated emission fluxes under low soil moisture, indicating adaptive defense prioritization under warm, dry conditions. Proteomic analyses reveal isoprene’s multifaceted protective roles: drought-modulated chloroplast protein abundance confirms its antioxidant activity, coupled with ABA biosynthesis regulation through hormone signaling crosstalk [207].

### 3.6. Mechanical Damage

Mechanical damage, as a typical physical stressor, reprograms VT emission profiles through multi-layered signaling networks. This response is mediated by rapid electrochemical signal propagation, which prioritizes VT metabolic activation over photosynthetic adjustments, coupled with oxidative stress and transcriptional cascades that drive dynamic VT synthesis. Isoprene metabolism shows particularly swift responsiveness to mechanical injury. Loreto and Sharkey [208] demonstrated that mechanical damage (cutting, abrasion) triggers immediate declines in isoprene emissions despite stable photosynthetic rates, indicating autonomous damage-sensing systems with heightened mechanosensitivity. Electrophysiological studies confirm that distal leaf tissues perceive damage via membrane potential shifts, suppressing *IspS* activity through long-distance electrical signaling. Disruption of membrane integrity and the consequent oxidative burst constitute core drivers of VOC dynamics. In mechanically wounded mango (*Mangifera indica*) branches, plasma membrane rupture induces ROS surges, elevating MVK and MACR concentrations by 0.8–0.9 ppbv and 0.3–0.6 ppbv, respectively, through lipid peroxidation [209,210]. This oxidative cascade is tightly coordinated with jasmonate signaling: mechanical stress activates LOX (lipoxygenase) and HPL (hydroperoxide lyase) genes, synchronously boosting precursor availability for stress-inducible VOC biosynthesis [211].

The spatiotemporal patterns of VT emissions manifest a dual-phase emission strategy encompassing immediate release and inducible biosynthesis. Constitutive (storage-type) VTs undergo rapid release upon mechanical disruption of secretory structures—exemplified by *Ageratina adenophora* releasing preformed amorpha-4, 7(11)-diene from laticifers via *EaTPS1*-mediated regulation [212], and freshly excised *Eucalyptus sideroxylon* leaves emitting dominant terpenoids (1,8-cineole, α-pinene, and limonene) from resin ducts [213]. Induced (synthesized de novo) VTs exhibit temporally phased biosynthesis, initiated by systemic signals like systemin that activate terpene synthase genes. Conifers subjected to wounding show progressive increases in xylem-emitted limonene and α-pinene under both light/dark cycles [214], demonstrating light-independent induction. This mechanical priming effect has agrotechnological relevance: in oolong tea production, the Yaoshiqing process (mechanical rocking) enhances key aroma compounds—linalool and geraniol concentrations increase 1.8–2.3-fold and 1.5–1.7-fold (reaching 38.7 ± 2.1 pmol g^−1^ h^−1^ and 25.3 ± 1.8 μg g^−1^ FW, respectively), collectively contributing over 67% of the characteristic floral notes. Mechanistically, wounding triggers rapid activation of ERF-family transcription factors, elevating their binding affinity to terpene synthase promoters (LIS, GES) by 3.2-fold. Concurrently, chromatin remodeling increases enhancer region accessibility (2.5-fold nucleosome displacement) at distal regulatory elements, potentiating transcriptional initiation efficiency of terpenoid biosynthetic genes [215].

Furthermore, mechanically induced VTs facilitate cross-individual defense networks through airborne signaling. For instance, herbivory by *Spodoptera littoralis* larvae induces a cascade effect in maize (*Zea mays*), where herbivore-induced plant volatiles (HIPVs) enhance terpenoid biosynthesis in both damaged and neighboring plants, strengthening systemic resistance against subsequent attacks [216]. In the field of agro-product processing, the epigenetic regulatory effects of mechanical damage warrant attention. During the shaking process of oolong tea, mechanical stress induces a decrease in DNA methylation levels within the promoter regions of terpene biosynthesis genes, which may sustain the upregulation of VOC production by alleviating gene silencing [215].

Mechanistic dissection reveals that mechanical stress orchestrates spatiotemporal precision in VT regulation through integrated electrical signaling, oxidative cascades, and transcriptional reprogramming. This systemic defense priming not only confers immediate stress resilience but also fosters chemically mediated ecological synergies, establishing cross-individual and cross-species defense coalitions. Comparative analyses of mechanotransduction pathways across plant taxa are essential for optimizing postharvest processing technologies and advancing next-generation stress-smart cultivars via pathway-specific metabolic engineering.

## 4. Discussion and Conclusions

The multifaceted roles of VTs in abiotic stress responses have increasingly positioned them as central components of plant adaptation strategies. Beyond their traditional classification as ecological signals or chemical defenses, VTs, particularly isoprene, monoterpenes (e.g., α-pinene and linalool), and sesquiterpenes (e.g., β-caryophyllene)—demonstrate clear antioxidant capabilities by directly quenching ROS and enhancing cellular antioxidant enzyme systems. These functions are essential for maintaining redox homeostasis under heat, drought, ozone, and light stress conditions. Such findings expand the current understanding of VTs as endogenous protectants that dynamically support both local cellular stability and systemic plant resilience. These stress-related functions are tightly coupled with well-characterized biosynthetic routes, namely the MVA and MEP pathways, and their regulation through transcription factors (e.g., MYC2, ERF, and WRKY), phytohormone interactions (JA, SA, and ABA), and enzymatic controls (e.g., DXS, DXR, HMGR, and TPS isoforms). The spatiotemporal flexibility of these regulatory nodes underpins the plasticity of VT biosynthesis and explains their responsiveness under compound stress environments. Given these insights, VTs offer several actionable avenues for crop improvement. Key regulators identified in this review—such as MYC2, TPSs, and DXR—are promising molecular targets for CRISPR-based editing or transgenic strategies aimed at enhancing multi-trait stress tolerance. Furthermore, selecting or engineering cultivars with enhanced capacity to emit or accumulate specific VTs may enable the development of stress-resilient genotypes with optimized redox buffering and reduced damage under extreme conditions. From a production standpoint, the capacity of VTs to act as intrinsic protectants opens up new perspectives for reducing input dependence in agriculture. Their potential as markers in breeding programs, foliar biostimulants, or bio-derived protective compounds aligns with the goals of climate-resilient and sustainable crop systems.

In conclusion, volatile terpenoids constitute a highly versatile and dynamic class of secondary metabolites that integrate environmental perception, metabolic regulation, and cellular protection in plants. This review synthesized recent advances in our understanding of VT biosynthesis, transcriptional and hormonal regulation, and abiotic stress responsiveness, with a particular focus on their emerging antioxidant functions. By bridging mechanistic knowledge with translational potential, we propose that key enzymes, transcription factors, and metabolic intermediates in VT pathways represent strategic entry points for improving plant performance under environmental constraints. Continued integration of multi-omics platforms and precision breeding tools will be pivotal in transforming VT-centered stress biology into actionable innovations for agriculture, enabling the next generation of stress-resilient crops and eco-compatible plant products. Looking ahead, several critical research directions can further advance our understanding and application of volatile terpenoids in plant stress adaptation and crop improvement. First, A critical avenue for future research involves elucidating the cascade mechanisms through which hormonal crosstalk regulates terpenoid biosynthesis. This issue is particularly relevant in long-lived woody species, where the temporal and spatial specificity of hormone signaling is closely linked to the maintenance of stress resilience over extended time scales. The ability of these plants to preserve metabolic plasticity and sustain energy balance under prolonged environmental stress is essential, especially in ornamental species and perennial crops, which require durable and coordinated regulatory systems. Second, the synergistic effects of multiple abiotic stressors, such as combined heat, drought, and high light, warrant deeper exploration. These compound stresses likely influence VT emission through resource-based constraints and signal interference among pathways (e.g., JA–SA antagonism), but such interactions remain poorly characterized. Third, the chemodiversity of terpenoids represents a largely untapped reservoir of functional traits. Moving beyond commonly studied volatiles such as pinene and limonene, future work should prioritize the identification of rare terpenes, the functional annotation of their biosynthetic enzymes, and the exploration of their ecological and physiological roles. These rare terpenoids may harbor unique antioxidant properties or stress-responsive functions and thus hold great promise for next-generation breeding strategies. Integrating high-resolution single-cell transcriptomics, spatial metabolomics, and real-time emission monitoring technologies will be instrumental in bridging the gene-to-function gap and facilitating the translation of volatile terpenoid research from mechanistic discovery to applied innovation in sustainable agriculture.

## Data Availability

No new data were created or analyzed in this study. Data sharing is not applicable to this article.
